# Supplementary material for: Cytological and Transcriptomic Analysis Provide Insights into the Formation of Variegated Leaves in Ilex × altaclerensis ‘Belgica Aurea’
Source: Plants (Basel). 2021 Mar 15;10(3):552. doi: 10.3390/plants10030552 (PMC7999392; doi:10.3390/plants10030552)
Supplement: Supplementary file 1 [file plants-10-00552-s001.zip › Supplementary files/Table S1.docx]

**Table S1.** An over view of the RNA-Seq data.

| **Sample** | **Total Raw Reads (M)** | **Total Clean Reads (M)** | **Total Clean Bases(Gb)** | **Clean Reads Q20(%)** | **Clean Reads Q30(%)** | **Clean Reads Ratio(%)** | **Total Mapping(%)** | **Uniquely Mapping(%)** |
| --- | --- | --- | --- | --- | --- | --- | --- | --- |
| **g1** | 71.96 | 68.69 | 6.87 | 97.63 | 89.78 | 95.46 | 87.13 | 24.95 |
| **g2** | 72.22 | 69.13 | 6.91 | 98.16 | 91.79 | 95.73 | 84.44 | 24.53 |
| **g3** | 69.73 | 66.74 | 6.67 | 98.08 | 91.49 | 95.71 | 83.36 | 25.13 |
| **v1** | 74.71 | 71.31 | 7.13 | 97.98 | 91.28 | 95.45 | 86.4 | 26.55 |
| **v2** | 64.08 | 61.39 | 6.14 | 97.63 | 89.65 | 95.8 | 87.07 | 28.04 |
| **v3** | 63.94 | 61.3 | 6.13 | 97.77 | 90.1 | 95.87 | 87.12 | 28.3 |
| **y1** | 74.45 | 70.9 | 7.09 | 97.63 | 89.72 | 95.22 | 87.34 | 27.47 |
| **y2** | 71.97 | 68.97 | 6.9 | 97.75 | 90.16 | 95.83 | 87.55 | 28.43 |
| **y3** | 69.45 | 66.01 | 6.6 | 97.59 | 89.66 | 95.06 | 87.05 | 28.2 |
