# Supplementary material for: Cytological and Transcriptomic Analysis Provide Insights into the Formation of Variegated Leaves in Ilex × altaclerensis ‘Belgica Aurea’
Source: Plants (Basel). 2021 Mar 15;10(3):552. doi: 10.3390/plants10030552 (PMC7999392; doi:10.3390/plants10030552)
Supplement: Supplementary file 1 [file plants-10-00552-s001.zip › Supplementary files/Table S9.docx]

**Table S9.** The primers for qRT-PCR analysis.

| **DEGs** | **Primer sequences** |
| --- | --- |
| CL374.Contig9-F | CGAGACTGTTGCGAGAACCT |
| CL374.Contig9-R | GCAGTGACCAAAGCAAGCAA |
| CL782.Contig2-F | GAGAGGCGATGAGGAGGAAC |
| CL782.Contig2-R | CCCAGTCTCAGGCTTGGTTT |
| CL1494.Contig2-F | CCCTCTGCAGTTCAACCCTT |
| CL1494.Contig2-R | GCATGTCCTGGGTCCTGATC |
| CL3869.Contig6-F | GGGTGGCAGCAGATAACCTT |
| CL3869.Contig6-R | AGAGAGAAGAATGGACAAGTGC |
| CL5332.Contig4-F | GGTTCTCATCCTGGCACCAA |
| CL5332.Contig4-R | ACCGACATCTCAAGGCCAAT |
| Unigene6311-F | AATGGAGTCTGGAGGGGTCA |
| Unigene6311-R | TGAGTGTCCTGATTGCTTTTGT |
| Unigene31820-F | TGAGGCGATTCGTGATGCAT |
| Unigene31820-R | GCCGGTGTACTCCTTCCAAA |
| Actin-F | TGGATAGCGACGTACATGGC |
| Actin-R | CCCAATCCTCCTGACCGAAG |
