# Supplementary figures and images for: Cytological and Transcriptomic Analysis Provide Insights into the Formation of Variegated Leaves in Ilex × altaclerensis ‘Belgica Aurea’
Source: Plants (Basel). 2021 Mar 15;10(3):552. doi: 10.3390/plants10030552 (PMC7999392; doi:10.3390/plants10030552)

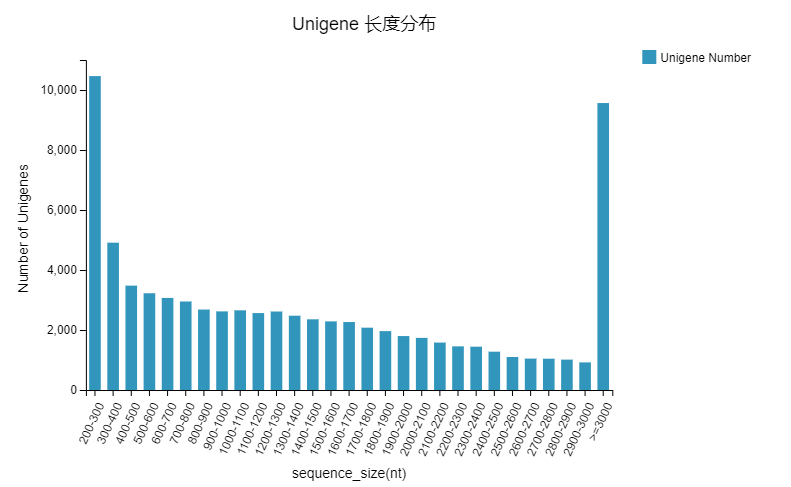


**Figure S1**

Supplement: Supplementary file 1 [file plants-10-00552-s001.zip › Supplementary files/Figure S1.docx]
